# The Niagara University—Medical Department

**Published:** 1883-11

**Authors:** 


### THE NIAGARA UNIVERSITY—MEDICAL DEPARTMENT.

This institution opened its first course of lectures at the Sisters of Charity Hospital, in this city, on the 10th of October, Prof. John Cronyn, the president of the faculty, delivering the introductory lecture. Notwithstanding the late issue to the profession of the announcements, and the higher standard of literary attainments required of its matriculants, the success of the new college exceeds the anticipation of its promoters, and is an augury that the profession are in earnest in their efforts for reform in the prevailing system of medical education.

The clinical advantages afforded by the large hospital now under the charge of the faculty of the college are ample, and the clinical instruction given the students under the guidance of the accomplished clinical professors are among the decided advantages the college offers to the profession for a thorough medical education. The graded curriculum and the annual examinations required in passing to the higher classes furnish, also, additional safeguards, as well as increased advantages. It is the aim of the faculty to submit the candidates for graduation to an independent board of examiners, thus practically endorsing the principles on which the Board of State Examiners is based, of divorcing the teaching and licensing power, and thus securing an impartial test for the qualifications of those who aspire to possess the degree of Doctor of Medicine.

We take pleasure in placing before the profession the evidence of success of an enterprise which inaugurates in this city a step in the direction of higher medical education; and we feel confident that ample encouragement will be given to enable the faculty to place the college on the same assured foundation that has made Harvard and other schools such a source of power and strength.

The class the present session is not large, but is is composed of students of excellent education and of earnest purpose. It is not the aim of the college to hold out inducements for the crowd. There are an abundance of opportunities elsewhere for cheap and easy medical education. The Niagara Medical College will give the students a thorough trial in all the branches laid down in its curriculum, and will send forth its graduates well equipped in practical details as well as in theoretical principles, so that the profession at large, and the public for whose benefit the profession labor, will realize the advantages of this and every other effort in the interests of higher medical education.

